# Plasma levels of neurogenic inflammation related neuropeptides in pediatric patients with community-acquired pneumonia and their potential diagnostic value in distinguishing viral and bacterial pneumonia

**DOI:** 10.1007/s00431-023-05417-y

**Published:** 2024-01-06

**Authors:** Mervan Bekdas, Bilgi Saygi, Yasemin Baranoglu Kilinc, Erkan Kilinc

**Affiliations:** 1 Department of Pediatrics, Faculty of Medicine, Bolu Abant Izzet Baysal University, Bolu, Turkey; 2 Department of Pediatrics, Bolu Izzet Baysal State Hospital, Bolu, Turkey; 3 Department of Physiology, Faculty of Medicine, Bolu Abant Izzet Baysal University, Bolu, Turkey

**Keywords:** Community-acquired pneumonia, Children, Neurogenic inflammation, Neuropeptides

## Abstract

Neurogenic inflammation is involved in the development and progression of respiratory inflammatory diseases. However, its role in community-acquired pneumonia (CAP) remains unclear. We therefore aimed to investigate plasma levels of neurogenic inflammation-related neuropeptides, calcitonin gene-related peptide (CGRP), substance P (SP), vasoactive intestinal peptide (VIP) and neuropeptide Y (NPY), and procalcitonin (PCT) in pediatric patients with CAP and to assess their diagnostic value in viral and bacterial/mixed pneumonia. A total of 124 pediatric patients with CAP (1 month-18 years old) and 56 healthy children of similar ages were prospectively enrolled. The patients were classified as viral (n = 99) and bacterial/mixed (n = 25) pneumonia. Plasma levels of the peptides were quantified by ELISA. ROC analysis was performed to evaluate possible diagnostic value of the peptides. While plasma levels of CGRP, VIP and PCT were significantly higher in patients with CAP than in the control group, respectively, NPY levels were significantly lower. Moreover, plasma levels of all neuropeptides and PCT were significantly higher in bacterial pneumonia patients compared to viral pneumonia patients. ROC analysis revealed that CGRP, SP and NPY had a diagnostic value in distinguishing viral and bacterial/mixed pneumonia.

*Conclusions*: Our findings suggest that these neuropeptides may be implicated in pediatric CAP. CGRP, SP and NPY together may be a promising candidate in distinguishing viral and bacterial/mixed pneumonia, however, for this, further studies are needed.

**Table:** 

**What is Known:** *• Neurogenic inflammation contributes to the development and progression of respiratory inflammatory diseases such as chronic obstructive pulmonary disease and bronchial asthma.*	
**What is New:** *• Plasma levels of neurogenic inflammation related neuropeptides calcitonin gene-related peptide, substance P, vasoactive intestinal peptide and neuropeptide Y are changed in pediatric community-acquired pneumonia. Calcitonin gene-related peptide, substance P and neuropeptide Y are promising candidates in distinguishing viral and bacterial/mixed pneumonia.*

**What is Known:**

*• Neurogenic inflammation contributes to the development and progression of respiratory inflammatory diseases such as chronic obstructive pulmonary disease and bronchial asthma.*

**What is New:**

*• Plasma levels of neurogenic inflammation related neuropeptides calcitonin gene-related peptide, substance P, vasoactive intestinal peptide and neuropeptide Y are changed in pediatric community-acquired pneumonia. Calcitonin gene-related peptide, substance P and neuropeptide Y are promising candidates in distinguishing viral and bacterial/mixed pneumonia.*

## Introduction

Community-acquired pneumonia in childhood is an important public health problem. Empirical antibiotic therapy targeting the most likely pathogens constitutes the basis of the management of bacterial community-acquired pneumonia [1], however, overuse of antibiotics has led to the development of antibiotic resistance, increased antibiotic-related complications and increased costs [2]. In order to prevent these undesirable results, an accurate differential diagnosis of bacterial and viral pneumonia should be made. However, an effective biomarker that can make this distinction is not yet known.

Increasing evidence suggests that there is a neuro-immune interaction in respiratory inflammatory diseases such as chronic obstructive pulmonary disease (COPD) and bronchial asthma [3]. In addition, neurogenic inflammation contributes to the development and progression of these types of respiratory conditions. Neurogenic inflammation is characterized by the release of the potent vasoactive neuropeptides calcitonin gene-related peptide (CGRP) and substance P (SP) from nociceptive sensory nerves, vasodilation, plasma extravasation, edema, and degranulation of immune mast cells. In addition to CGRP and SP, other vasoactive neuropeptides released from nerves innervating respiratory system structures, vasoactive intestinal peptide (VIP) and neuropeptide Y (NPY) also modulate neurogenic inflammation [4].

Viruses and bacteria that penetrate into the respiratory tract are capable of directly stimulating nociceptors/sensory nerves [5, 6]. The activated nociceptors/sensory nerves in turn release the neuropeptides through axon reflex, resulting in neurogenic inflammation [7]. However, the role of neurogenic inflammation and related neuropeptides in the pathogenesis of viral and bacterial pneumonia remains unclear.

We therefore aimed to investigate the plasma levels of these neurogenic inflammation-related neuropeptides and procalcitonin (PCT), a promising biomarker for the diagnosis of bacterial infections, in pediatric patients with community-acquired pneumonia. We also explored whether these neuropeptides have diagnostic value in differentiating viral and bacterial/mixed pneumonia.

## Materials and methods

### Participants

A total of 124 patients aged 1 month-8 years who were hospitalized with the diagnosis of community-acquired pneumonia in the pediatric service of our hospital between February 2022 and January 2023 were prospectively included in the study. Additionally, 56 healthy children aged 1 month-8 years were included in the control group. Children in the control group were chosen from healthy volunteers who applied to the hospital for check-ups for school and sports activities, had normal examination findings and no any disease. In keeping with the Declaration of Helsinki, healthy controls, patients and their parents released their informed consents. All patients and healthy volunteers were of Turkish origin and from the Western Black Sea region of Turkey. The study protocol was approved by the Bolu Abant Izzet Baysal University Clinical Research Ethics Committee, Bolu, Turkey (license number 2022/04) and was conducted in accordance with Good Clinical Practices and the Declaration of Helsinki.

Inclusion criteria for the patients were: be older than 1 month and less than 18 years old, not have any chronic disease, have been diagnosed with community-acquired pneumonia. Exclusion criteria for the patients were: those diagnosed with mild pneumonia, those with any chronic neuro-inflammatory disease, those who have had an infectious disease other than pneumonia, those with immunodeficiency, those who take immunosuppressant drugs or have any mast cell disease such as mastocytosis. The demographic characteristics of the all volunteers such as age, gender, weight and height were recorded. In addition, using an elaborative questionnaire, the features of the patients such as complaints at the time of admission (fever, cough, chest pain, wheezing, respiratory distress), physical examination findings (fever, cyanosis, respiratory rate, pulse, oxygen saturation value, rale, rhonchi, retraction, wheezing), length of hospitalization and admission to the intensive care unit were recorded.

A peripheral vascular access was established for the hospitalized patients, during which complete blood count, peripheral smear, C-reactive protein (CRP), erythrocyte sedimentation rate and blood culture were requested. Moreover, samples were taken for evaluation of coronavirus disease-19 by PCR. In order to confirm pneumonia, chest radiographs were taken at the time of admission to all patients and pathological features used in the definition of pneumonia (such as infiltration, pneumatocele, atelectasis, increased aeration) and accompanying complications (pleural effusion, pneumothorax etc.) were noted. Community-acquired pneumonia was diagnosed based on the symptoms of respiratory system infection, pathological findings on auscultation, and the presence of lung infiltration findings on the chest x-ray [8]. None of our cases died.

### Classification of pneumonia cases as viral or bacterial/mixed

Patients were classified into viral and bacterial/mixed pneumonia based on serum levels of PCT, neutrophil counts and CRP values. Because it is well documented that serum levels of CRP and PCT, and neutrophil counts were higher in bacterial/mixed cases than viral cases in pediatric pneumonia although they are not yet used in clinical routine [9–11]. Moreover, it has previously been suggested that pneumonias with serum PCT > 800 pg/ml (= 0.8 ng/ml) can be classified as bacterial/mixed, while those with PCT < 800 pg/ml can be classified as viral [12]. Accordingly, cases that had all three features below were evaluated as bacterial/mixed: i) serum PCT > 800 pg/ml, ii) higher serum CRP than others, and iii) higher neutrophil counts than others. The remained cases were considered as viral pneumonia.

### Collection of blood samples

During the hospitalization, approximately 2 ml of venous blood was collected from each patient, except for routine examinations, while peripheral vascular access was established. Likewise, venous blood was collected from healthy volunteers. The blood samples were immediately put into ice-cold glass tubes containing the anticoagulant EDTA and 100 μL of protease inhibitor cocktail (cOmplete, Sigma-Aldrich) to avoid the degradation of the peptides and centrifuged at 4000 rpm for 10 min at 4 °C. The supernatant samples were stored at -20 °C until being assayed for CGRP, SP, VIP, NPY and PCT immunoreactivities using the enzyme-linked immunosorbent assay (ELISA) method.

### Measurement of plasma levels of CGRP, SP, VIP, NPY and PCT

Concentrations of CGRP, SP, VIP, NPY and PCT in the supernatant samples were determined using ELISA kits (BT Lab, Shanghai, China). Assay procedures were performed in accordance with the manufacturer’s instructions and in duplicate. Shortly, 50 µL of CGRP, SP, VIP, NPY and PCT standard and 40 µl of supernatant samples was put to the relevant wells. Thereafter, 50 μl of streptavidin-HRP was put to each well of 96 well plate. After the incubation and washing, 50 μl of solution-A and solution-B were put to all wells, respectively. After the incubation, 50 μl of stop solution was added to all wells. The optical density was quantified at 450 nm by a microplate reader (Epoch BioTek Instruments Inc., Winooski, VT, USA). The peptide concentrations were calculated.

### Statistical analysis

The data obtained from the groups were expressed as mean ± standard deviation. SPSS software (Version 22.0, IBM Corp., NY, USA) was used to analyze the data. Normality analysis of the data was carried out with Kolmogorov–Smirnov test. Since the data did not exhibit a normal distribution, while multiple comparisons were performed with the Kruskal–Wallis followed by Dunn's multiple comparison post-hoc test, the difference between the two groups was analyzed with the Mann Whitney-U test. The diagnostic value of the peptides were evaluated by the receiver operating characteristic curve (ROC) analysis. *P* < 0.05 was considered as significant.

## Results

### Demographic attributes of patients with general pneumonia and healthy controls

There was no significant difference in terms of gender and age of patients with general pneumonia compared to healthy controls (p = 0.079 and p = 0.57, respectively, Table 1).

**Table 1:** Demographic attributes of patients with pneumonia and healthy controls

	Gender		Age (years)	Hospitalization		Total number of subjects
	Male	Female		Number (percent)	Days	
General *pneumonia*	71 (57.2%)	53 (42.7%)	5.6 ± 4.3	5 (4.0%)	3.4 ± 2.6	124
Bacterial/mixed	16 (64%)	9 (36%)	5.9 ± 3.1	2 (8.0%)	3.4 ± 2.4	25
Viral	57 (57.6%)	42 (42.4%)	5.5 ± 4.5	3 (3.0%)	3.4 ± 2.7	99
Healthy controls	26 (46.4%)	30 (53.6%)	5.5 ± 2.4	-	-	56

### A complete blood count parameters of patients with general pneumonia and healthy controls

WBC and neutrophil counts were higher in individuals with pneumonia compared to the control group (13231 ± 6299 vs 8008 ± 2515/mm^3^, *p* < 0.001 and 9297 ± 5586 vs 3942 ± 1807/mm^3^, *p* < 0.001, respectively). Likewise, CRP and erythrocyte sedimentation values in individuals with pneumonia were also significantly higher than that in the control group (43.8 ± 51.2 vs 0.4 ± 1 mg/dL, *p* < 0.001 and 33.3 ± 22.7 vs 12 ± 2.8 mm/h, p = 0.033). There was no significant difference between the groups for other biochemical parameters (*p* > 0.05).

### Demographic attributes of patients with pneumonia subgroups

There was no significant difference between patients with bacterial/mixed pneumonia and viral pneumonia in terms of age and sex (p = 0.212, p = 0.652, respectively, Table 1). Moreover, there was no significant difference between the groups in terms of previous hospitalization rates for pneumonia and hospitalization times (p = 0.25, (p = 0.43, respectively, Table 1).

### A complete blood count parameters of patients with pneumonia subgroups

Neutrophil counts were significantly higher in individuals with bacterial/mixed pneumonia compared to those with viral pneumonia (11461 ± 6095 vs. 8751 ± 5345 /mm3, p = 0.026). Likewise, CRP values were significantly higher in those with bacterial/mixed pneumonia compared to those with viral pneumonia (60.8 ± 59.9 vs 39.5 ± 48.1 mg/dL, p = 0.016). On the other hand, there was no significant difference between the groups in terms of other biochemical parameters (*p* > 0.05).

### Plasma levels of neuropeptides and procalcitonin in general pneumonia and control groups

**Fig. 1:**
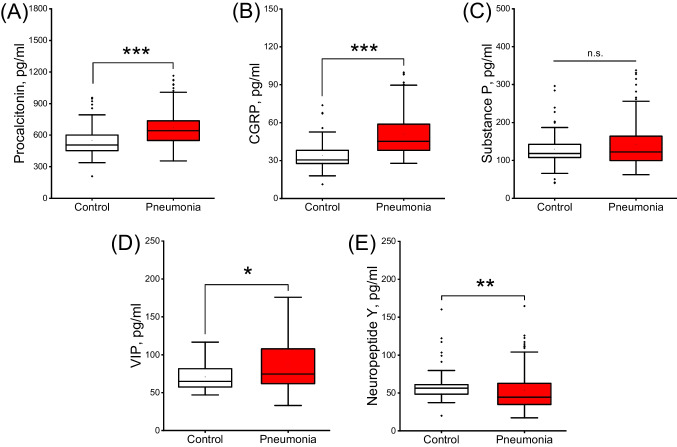
Plasma levels of **A** procalcitonin, **B**, calcitonin gene-related peptide, **C**, substance P, **D**, vasoactive intestinal peptide, **E**, neuropeptide Y in the pneumonia and healthy control groups. Each box represents the median values, and first and third quartiles of the results. The pneumonia group (n = 124) represents all pediatric patients with pneumonia in the study, regardless of whether they are bacterial or viral pneumonia. Plasma levels of these peptides between pneumonia (n = 124) and control (n = 56) groups are compared by Mann–Whitney U test. CGRP, calcitonin gene-related peptide; VIP, vasoactive intestinal peptide; n.s., non-significance. *p < 0.05, **p < 0.01 and ***p < 0.001

Plasma level of PCT was significantly higher in individuals with pneumonia than in the control group (*p* < 0.001, Fig. 1A). Likewise, plasma levels of CGRP and VIP were significantly higher in patients with pneumonia than in the control group (*p* < 0.001, Fig. 1B and p = 0.011, Fig. 1D respectively). In contrast, plasma level of NPY was significantly lower in patients with pneumonia than in the control group (p = 0.002, Fig. 1E). On the other hand, there was no significant difference between the groups in terms of plasma levels of SP (p = 0.14, Fig. 1C).

### Plasma levels of neuropeptides and procalcitonin in pneumonia subgroups and control group

**Fig. 2:**
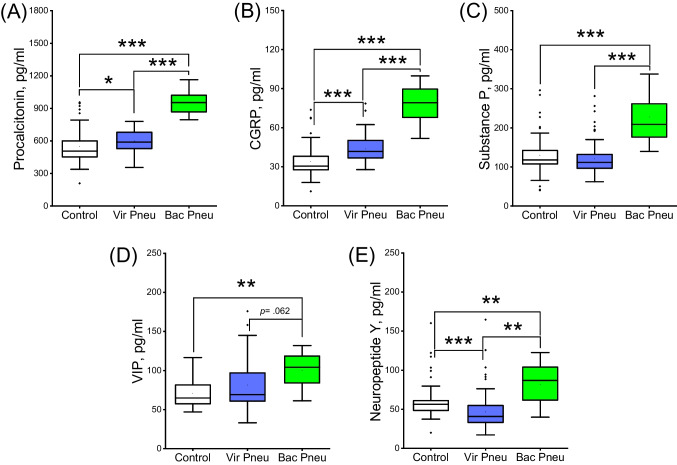
Plasma levels of **A** procalcitonin, **B**, calcitonin gene-related peptide, **C**, substance P, **D**, vasoactive intestinal peptide, **E**, neuropeptide Y in the pneumonia subgroups and healthy control group. Each box represents the median values, and first and third quartiles of the results. Bacterial pneumonia (n = 25), viral pneumonia (n = 99) and control (n = 56) groups were analyzed by Kruskal–Wallis test followed by Dunn’s multiple comparison test. CGRP, calcitonin gene-related peptide; VIP, vasoactive intestinal peptide. *p < 0.05, **p < 0.01 and ***p < 0.001

Plasma levels of PCT were significantly higher in patients with both bacterial/mixed and viral pneumonia compared to the control group (*p* < 0.001 and p = 0.035, Fig. 2A). Moreover, plasma levels of PCT in patients with bacterial/mixed pneumonia were significantly higher than that in viral pneumonia (*p* < 0.001, Fig. 2A).

Plasma levels of CGRP were significantly higher in patients with both bacterial/mixed and viral pneumonia compared to the control group (*p* < 0.001 and *p* < 0.001, Fig. 2B). Moreover, plasma levels of CGRP in patients with bacterial/mixed pneumonia were significantly higher than that in viral pneumonia (*p* < 0.001, Fig. 2B).

Plasma levels of SP and VIP were significantly higher in patients with bacterial/mixed pneumonia compared to the control group (*p* < 0.001, Fig. 2C for SP; p = 0.002, Fig. 2D for VIP) while there is no significant difference between viral pneumonia and control groups respectively (p = 0.734, Fig. 2C for SP; p = 0.152, Fig. 2D for VIP). In addition, plasma levels of SP in patients with bacterial/mixed pneumonia were significantly higher than that in viral pneumonia (*p* < 0.001, Fig. 2C). On the other hand, there was no significant difference between the groups in terms of plasma levels of VIP (p = 0.062, Fig. 2D).

Plasma levels of NPY were significantly higher in patients with bacterial/mixed compared to both the control and viral pneumonia groups, respectively (p = 0.001 and p = 0.001, Fig. 2E). However, plasma levels of NPY were significantly lower in patients with viral pneumonia compared to the control group (*p* < 0.001, Fig. 2E).

### The diagnostic value of neuropeptides and procalcitonin in differentiating viral and bacterial/mixed pneumonia

**Fig. 3:**
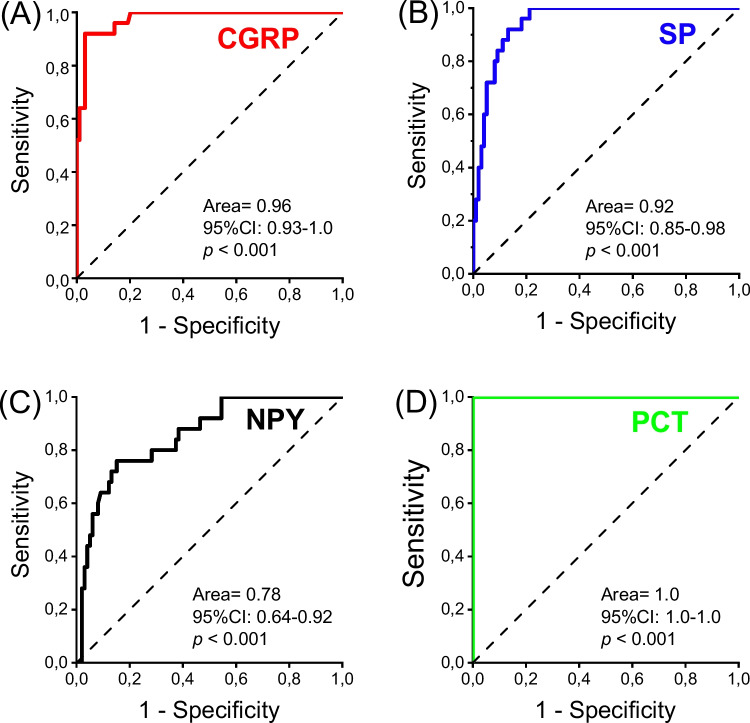
ROC curve analysis of plasma (**A**), calcitonin gene-related peptide (**B**), substance P, (**C**), neuropeptide Y, (**D**), procalcitonin in differentiating bacterial pneumonia and viral pneumonia. CGRP, calcitonin gene-related peptide; SP, substance P; NPY, neuropeptide Y; ROC, receiver operating characteristic

The ROC analysis revealed CGRP, SP, NPY and PCT had a diagnostic value in differentiating viral and bacterial/mixed pneumonia (AUC [95% CI]: CGRP, 0.96 [0.93–1.0], p < 0.001; SP, 0.92 [0.85–0.98], p < 0.001; NPY, 0.78 [0.64–0.92], p < 0.001; PCT, 1.0 [1.0–1.0], p < 0.001, Fig. 3A–D).

## Discussion

It is very important to determine the cases with pneumonia in which antibiotics will be used, because the delay in starting antibiotics increases the risk of sepsis and respiratory failure. However, unnecessary use of antibiotics in viral pneumonia leads to an increase in antibiotic resistance in bacteria. The lack of rapid and accurate tests to distinguish between viral and bacterial/mixed respiratory infections is the biggest obstacle to optimizing antibiotic use. It is of great importance to investigate candidate tools that can make this distinction.

It was demonstrated that pneumonia patients with bacterial/mixed origin have higher levels of WBC and CRP compared to those of atypical or viral origin [13]. In line with this, we found that WBC, neutrophil, CRP and erythrocyte sedimentation values were increased in those with bacterial/mixed pneumonia compared to those with viral origin.

Neurogenic inflammation participates in the pathobiology of many diseases such as migraine, asthma, fibromyalgia, atopic dermatitis, psoriasis. There is a relationship between vasoactive neuropeptides-mediated neurogenic inflammation and respiratory inflammatory diseases such as bronchial asthma and COPD [4]. However, most of the evidence for this relationship comes from preclinical studies, and little is known about the role of neurogenic inflammation in humans. On the other hand, there is no study yet on the role of neurogenic inflammation in pathological processes induced by pneumonia. The present study revealed for the first time that plasma levels of neurogenic inflammation-related CGRP and VIP neuropeptides increased and NPY levels decreased in pediatric patients with pneumonia. However, the plasma levels of the SP remained unchanged. In addition, plasma level of PCT, a promising biomarker for the diagnosis of bacterial infections, also increased in the patients with pneumonia. Moreover, plasma levels of all neuropeptides and PCT in patients with bacterial/mixed pneumonia were significantly higher than that in patients with viral pneumonia.

Of neuropeptides related to neurogenic inflammation, CGRP and SP are released from sensory fibers innervating the respiratory system, VIP from parasympathetic and sensory fibers, and NPY from sympathetic fibers [4]. The release of these neuropeptides from the respiratory nerves can be induced by house dust mites, viruses, bacteria, inflammatory mediators and irritants [14]. Moreover, CGRP, SP and VIP contribute to neurogenic inflammation by inducing activation of immune mast cells that are early responders of inflammatory processes [7].

CGRP and SP induced development of neurogenic inflammation during infection [3]. Moreover, CGRP levels were increased in patients with sepsis [15]. CGRP levels in sputum and counts of CGRP-positive cells in the airways were raised in patients with COPD and asthma [16, 17]. We found that CGRP levels were increased in pediatric patients with both bacterial and viral pneumonia. Our findings are consistent with previous studies and suggest that CGRP may have a role in neurogenic inflammation in pneumonia.

On the other hand, VIP is one of the most abundant neuropeptides in the upper and lower respiratory tract in human and considered as anti-inflammatory [4, 18]. Serum levels of VIP were higher in acute exacerbation of COPD than in stable COPD and VIP decreased cigarette smoke-stimulated injury of alveolar cells and apoptosis [19]. VIP levels, like anti-inflammatory cytokines, appear to increase to reduce/prevent damage in inflammatory conditions. Considering the vasodilator and mast cell activating features of VIP, it may be suggested that it has a dual role. We found that VIP levels were increased in pediatric patients with bacterial pneumonia, but not in patients with viral pneumonia, compared to healthy controls. This may be a sign that VIP may come into prominence in bacterial pneumonia rather than viral pneumonia.

SP is involved in inflammatory diseases of the respiratory system. Patients with asthma are over-responsive to SP and SP leads to bronchoconstriction, increased mucus secretion and plasma leakage [20]. We found that SP levels were elevated in pediatric patients with bacterial pneumonia, but not in patients with viral pneumonia, compared to healthy controls while it did not change in pediatric patients with general pneumonia. These findings evidently suggest that the increase in SP levels may be specific to bacterial pneumonia rather than viral pneumonia. In addition, elevated circulating SP levels suggest that it has a role in neurogenic inflammatory processes induced by bacterial pneumonia.

It has been suggested that PCT may be a promising biomarker in diagnosis of bacterial infections and in distinguishing bacterial pneumonia and viral pneumonia [21, 22]. We found that PCT levels were increased in patients with both bacterial/mixed and viral pneumonia. This indicates that increased PCT levels are important in the diagnosis of community-acquired pneumonia. However, it was previously suggested that PCT alone may not be sufficient to distinguish bacterial infections and viral infections [23]. More clinical studies are needed on whether PCT alone can be used as a tool in differentiating bacterial pneumonia and viral pneumonia.

Unlike the other neuropeptides, we found that NPY levels were lower in patients with general pneumonia compared to the healthy controls. When analyzed according to pneumonia subgroups, it is understood that this decrease in NPY levels is due to patients with viral pneumonia. Because, NPY levels were higher in patients with bacterial pneumonia compared to healthy controls, while it was lower in patients with viral pneumonia. It is reported in the literature that NYP levels are mostly increased in inflammatory conditions [24–26]. Our findings regarding increased NPY levels in bacterial pneumonia are consistent with this previous literature and imply a role of NPY in pathological processes in bacterial pneumonia.

The increase in NPY levels may also be for the purpose of preventing/reducing inflammatory damage caused by bacterial pneumonia. Because there are also studies in the literature reporting the anti-inflammatory effects of NPY. Unlike the other neuropeptides (CGRP, SP and VIP), NPY is a vasoconstrictor and also promotes angiogenesis [27] and blocked generation of pulmonary fibrosis through inhibition of IL-1β [28]. Neuropeptide Y can exert both pro-inflammatory and anti-inflammatory effects depending on the cell or receptor it acts on [29]. It therefore has a dual role in inflammatory processes.

It is interesting that NPY levels were lower in patients with viral pneumonia in the current study. Publications on the association of low NPY levels with diseases are rare [30, 31]. A study reported that when acute kidney injury develops, serum neuropeptide Y levels decrease rapidly in the early stage, and then it can protect the kidney by inhibiting macrophage activation [31]. In the current study, the decrease in neuropeptide Y levels in viral pneumonia cases may be related to the decrease in this early stage. More studies need to clarify the reasons for the decrease in NPY levels in pediatric patients with viral pneumonia.

In the current study, besides elevated levels of CGRP, SP, VIP, NPY, and PCT peptides in bacterial pneumonia compared to controls, these were also all higher in bacterial pneumonia than in viral pneumonia. Moreover, the ROC analysis revealed that CGRP, SP, NPY and PCT, except for VIP, had a diagnostic value in distinguishing viral and bacterial/mixed pneumonia. Although the relatively small sample size and single-center nature of our study can limit the generalizability of the findings, these peptides may represent diagnostic biomarkers to distinguish between bacterial and viral pneumonia. Future multicenter studies with a large sample size can further contribute to the potential of these peptides to be used as a differential diagnostic marker.

## Limitations of the study

The fact that the study was single-centered and the sample size was relatively small may limit the generalizability of the findings. The classification of pneumonia cases was made based on the patients' serum PCT and CRP levels and neutrophil counts. This may weaken the implications about diagnostic value of the neuropeptides in differentiating bacterial/mixed and viral pneumonia. Future studies with a microbiological, serological or PCR-based classification could further promote the findings about the diagnostic value of the neuropeptides.

## Conclusions

Taken together, our findings suggest that neurogenic inflammation-related neuropeptides CGRP, SP, VIP and NPY may be involved in pathological processes in pediatric community-acquired pneumonia. CGRP, SP and NPY together may be a promising tool in distinguishing viral and bacterial/mixed pneumonia and in monitoring disease progression and treatment response in children, however, for this, further studies are needed.

## Data Availability

Data are available from the corresponding author on reasonable request.

## Abbreviations

CGRP: Calcitonin gene-related peptide
COPD: Chronic obstructive pulmonary disease
CRP: C-reactive protein
IL-1β: Interleukin-1 beta
NPY: Neuropeptide Y
PCT: Procalcitonin
ROC: Receiver operating characteristic curve
SP: Substance P
VIP: Vasoactive intestinal peptide
WBC: White blood cell
